# Effect of freeze–thaw cycles on the nutritional quality of some selected Nigerian soups

**DOI:** 10.1002/fsn3.271

**Published:** 2015-08-13

**Authors:** Akeem Olayemi Raji, Rahman Akinoso, Monsurat Oyewale Raji

**Affiliations:** ^1^ Department of Food Agric and Biological EngineeringCollege of Engineering and TechnologyKwara State UniversityMaleteIlorinNigeria; ^2^ Department of Food TechnologyUniversity of IbadanIbadanOyo StateNigeria

**Keywords:** Freezing, nutritional quality, soups, thawing

## Abstract

Freezing and thawing are heat transfer processes, involving chemical changes which may greatly affect product quality. Due to sparse literature on freeze–thawed cycles and its effects on soups quality, the effect of freeze–thaw cycles on the nutritional quality of selected Nigerian soups has to be investigated. Soups (*Ila*,* Ewedu*,* Ogbono*, and *Kuka*) were prepared using standard recipes. The soups were packaged in plastic and aluminum containers, frozen at −20°C, and thawed with microwave oven, hot water (100°C), and at ambient condition for four cycles of 5‐day interval. After each cycle, chemical compositions of the samples were determined using AOAC methods. Data were analyzed using ANOVA at *P* = 0.05. Moisture, protein, fat, crude fiber, ash, and carbohydrate contents of the freeze–thawed soups were 63.6–88.6%, 3.6–8.8%, 1.0–6.1%, 0.8–1.2%, 1.8–4.6%, and 0.9–15.6%, respectively. Mineral contents were iron (5.0–6.8 mg/100 g), calcium (68.1–190.8 mg/100 g), sodium (144.4–231.7 mg/100 g), potassium (200.4–302.1 mg/100 g), and phosphorus (228.0–337.2 mg/100 g). Vitamins were vitamin A (29.5–59.9 mg/100 g), vitamin B (10.1–36.4 mg/100 g), and vitamin E (28.4–90.2 mg/100 g). Microwave‐thawed plastic soups had limited nutritional losses when compared with other thawing methods, and should not be extended beyond the third cycle because of increasing reduction in fat and protein, indicating deterioration.

## Introduction

Leafy vegetables are important in many Nigerian diets. Apart from the variety which they add to the menu, they are valuable sources of mineral, vitamins, fiber, and other nutrients which are usually in short supply in daily diets (Mepba et al. 2007). Also, they contribute to the flavor, taste, color, and esthetic appeal to what would otherwise be a monotonous diet.

Nutrients are essential for physical growth, maintenance of normal body function, and good health. Nutrition is a basic prerequisite for life sustenance (Soundarapandian et al. 2013). Minerals constitute the micronutrients and they are necessary for physiological and biochemical processes by which the human body acquires, assimilates, and utilized food to maintain health and activity (Mohapatra et al. 2009). Minerals do not only promote proper physical growth and development, but also ensure adequate immune competence and cognitive development (Soundarapandian et al. 2013). Although vegetables are highly nutritious, yet they are perishable (Hart et al. 2005). Preservation of vegetables and vegetable‐related products by freezing to prevent nutritional losses is important.

The use of the freezing process to increase the length of food has gained widespread attention since the reduction in available water due to the formation of ice crystals and subzero temperatures provides an environment which favors reduced chemical reactions leading to increased storage stability (Zaritzky 2006). However, freezing is not a perfect method of preservation since even at low temperatures food quality deterioration may still occur. The formation of ice can result in textural changes and disruption of cell compartments causing the release of chemically reactive components (Lim et al. 2004). Thawing is in fact a temperature abuse, and has to be considered a critical operation in terms of quality and safety. Food safety has been addressed for several thawing methods (Yamamoto and Harris 2001), whereas quality losses are not generally tackled by the scientific community. Nutritional losses that occur during thawing decrease the health benefits of frozen green vegetables included into a diet.

Traditionally, soups are generally reheated several times in the day to prevent spoilage. This method of vegetable utilization has been shown to result in losses of vitamins (Hart et al. 2005). Such a situation calls for proper preservation and utilization of vegetables for maximum nutritional benefits. In modern homes, most soups are kept under frozen condition to preserve them and to avoid nutritional loses which may arise as a result of periodical heating after use (Hart et al. 2005). These freeze–thaw cycles may be repeated several times and it is very important to determine the quality changes that occur during multiple freezing–thawing treatments. The changes induced by the freezing–thawing cycle are mainly due to three phenomena that are often closely related: mechanical damage, denaturation of proteins, and loss of water‐holding capacity (Hallier et al. 2007).

The standard methods of preparing various Nigerian soups and stews had been established by FIIRO (2006), but little has been done about their preservation and on various preservation techniques that they are subjected to. Freezing and thawing processes are complex, involving heat transfer and possibilities of a series of physical and chemical changes which may greatly affect product quality. From quality point of view, the effect of freeze–thaw cycles on the nutritional quality of selected Nigerian soups has to be investigated.

## Material and Methods

### Materials

The ingredients used for the preparation of the above soups were purchased from local markets at Ipata and Ago in Ilorin, Kwara State, Nigeria.

### Methods

#### Preparation of soups

Selected Nigerian soups (*Ewedu*,* Ila*,* Ogbono*, and *Kuka*) were prepared using facilities of the Department of Food, Agricultural, and Biological Engineering, Kwara State University, Nigeria. The preparation methods used for the selected soups were those earlier established by recipe book of the Federal Institute of Industrial Research, Oshodi (FIIRO, 2006). The recipes used for the soups were presented in Table 1 and the methods of preparation were described later.

**Table 1:** Recipes used for preparation of soups

*Ogbono*	*Ewedu*	*Ila*	*Kuka*
Bitter leaf (20 g)	Leaves (120 g)	Okro (fruit – 640 g)	Meat (1000 g)
*Ogbono* (ground – 240 g)	Egusi (ground – 40 g)	Pepper (14 g)	Onion (75 g)
Palm oil (80 mL)	Water (500 mL)	Ugwu (200 g)	Dry fish (50 g)
Water (2000 mL)	Crayfish (40 g)	Meat (1000 g)	Stockfish (500 g)
Maggi (8 g)	Potash (1 g)	Crayfish (40 g)	*Kuka* (Powder 150 g)
Onion (3400 g)	Iru (5 g)	Onion (300 g)	Maggi (10 g)
Iru (locust beans – 10 g)	Salt (5 g)	Salt (14 g)	Salt (8 g)
Fish (smoked – 310 g)		Palm oil (40 mL)	Curry (3 g)
Meat (1000 g)		Water (2000 mL)	Thyme (1.4 g)
Crayfish (ground – 40 g)		Maggie cube (8 g)	Crayfish (30 g)
Periwinkle (deshelled – 94 g)		Iru (locust beans – 10 g)	Water (2000 mL)
Salt (10 g)			Palm oil (100 mL)
Pepper (ground – 14 g)			

Adapted from FIIRO (2006) methods.

#### Freezing and thawing of soups

A laboratory scale chest freezer (Scanfrost chest; Model SFL‐111, Hangzhou, China) with natural convection at −20°C was used to freeze and store the soup samples. The frozen soups were stored at −20°C for 5 days before being thawed. Three different thawing methods were employed.

1. Thawing in a microwave oven (LG, MS2024W, using defrost program, 450 W max. power)
2. Thawing in hot water (100°C) using water bath
3. Thawing at ambient temperature (28–32°C)

However, the products were thawed until the temperature at the center of the soups reached 0°C and this was ensured using a digital thermometer. Parts of the thawed packed soups were placed immediately on ice for analyses (cycle 1). In order to imitate thawing and refreezing that frozen soups experienced when some portions are consumed in modern homes, the other packed soups were frozen in a still freezer at −20°C for 5 days and thawed using the thawing methods stated earlier. Parts of the thawed soups were also placed on ice for analysis (cycle 2), while the rest were frozen. The freeze–thaw step (5 days storage at −20°C and then thawed) were repeated for four cycles.

### Analyses

The proximate composition (moisture, protein, ash, fat, crude fiber, and carbohydrate), mineral (iron, calcium, sodium, potassium, and phosphorus), and vitamin contents (vitamin A [retinol], vitamin B [thiamine], and vitamin E) of the soup samples were evaluated using the standard AOAC procedure (AOAC, 2005). Data were expressed as mean ± SD and were analyzed by one‐way ANOVA test using SPSS statistical programme.

## Results and Discussion

### Effect of freezing and thawing conditions on proximate composition of some selected Nigerian soups

Tables 2, 3, 4, 5 present the effect of freezing and thawing conditions on proximate composition of *Ogbono*,* Ewedu*,* Ila*, and *Kuka* soups subjected to frozen storage. There were significant differences (*P* < 0.05) in the proximate composition of *Ogbono*,* Ewedu*,* Ila*, and *Kuka* soups when subjected to the above frozen and thawing conditions as compared to the freshly prepared *Ogbono*,* Ewedu*,* Ila*, and *Kuka* soups, except for the moisture content of *Ewedu* soup samples and crude fiber of *Ewedu* and *Ogbono* soup samples that were not significantly different.

**Table 2:** Effect of freezing and thawing conditions on proximate composition of *Ewedu* soups

Samples	%Moisture	%Protein	%Fat	%Ash	%Crude fiber	%Carbohydrate
Cycle 0						
E	88.60 ± 0.14^a^	6.00 ± 0.01^a^	1.05 ± 0.05^a^	1.81 ± 0.01^j^	1.04 ± 0.60^a^	7.41 ± 0.20^n^
Cycle 1						
AH	88.07 ± 0.03^a^	5.77 ± 0.15^a–c^	1.04 ± 0.04^a^	1.84 ± 0.02^g–j^	1.02 ± 0.60^a^	8.25 ± 0.06^kl^
AR	87.46 ± 0.05^a^	4.78 ± 0.24^f^	1.03 ± 0.04^a^	1.96 ± 0.05^e^	1.00 ± 0.60^a^	9.78 ± 0.42^g^
AM	87.35 ± 0.14^a^	5.84 ± 0.12^ab^	1.04 ± 0.03^a^	1.83 ± 0.02^h–j^	1.02 ± 0.60^a^	8.61 ± 0.07j
PH	87.66 ± 0.05^a^	5.87 ± 0.12^ab^	1.05 ± 0.03^a^	1.83 ± 0.02^ij^	1.03 ± 0.59^a^	7.85 ± 0.12^m^
PR	87.78 ± 0.08^a^	4.60 ± 0.10^fg^	1.03 ± 0.03^a^	1.99 ± 0.01^c–e^	0.98 ± 0.61^a^	9.06 ± 0.03^i^
PM	88.48 ± 0.03^a^	5.92 ± 0.07^ab^	1.05 ± 0.05^a^	1.81 ± 0.01^j^	1.03 ± 0.61^a^	8.10 ± 0.10^l^
Cycle 2						
AH	87.46 ± 0.05^a^	5.50 ± 0.10^c–e^	1.00 ± 0.09^a^	1.86 ± 0.02^f–j^	1.01 ± 0.60^a^	9.05 ± 0.12^i^
AR	86.78 ± 0.71^a^	4.37 ± 0.31^gh^	0.95 ± 0.13^a^	1.99 ± 0.01^de^	1.01 ± 0.60^a^	11.49 ± 0.09^d^
AM	85.79 ± 0.09^a^	5.60 ± 0.10^b–d^	1.02 ± 0.07^a^	1.85 ± 0.02^f–j^	1.01 ± 0.60^a^	9.68 ± 0.12^g^
PH	86.72 ± 0.03^a^	5.60 ± 0.10^b–d^	1.03 ± 0.07^a^	1.84 ± 0.01^g–j^	1.02 ± 0.59^a^	8.33 ± 0.10^k^
PR	87.06 ± 0.09^a^	4.27 ± 0.31^h^	0.95 ± 0.13^a^	2.04 ± 0.05^a–c^	0.98 ± 0.61a	10.51 ± 0.10^e^
PM	88.40 ± 0.05^a^	5.80 ± 0.05^ab^	1.05 ± 0.05^a^	1.82 ± 0.02^j^	1.02 ± 0.62^a^	8.77 ± 0.09^j^
Cycle 3						
AH	86.90 ± 0.11^a^	5.20 ± 0.20^e^	1.00 ± 0.09^a^	1.89 ± 0.03^fg^	0.99 ± 0.60^a^	9.83 ± 0.12^g^
AR	85.31 ± 0.09^a^	3.90 + 0.20^i^	1.00 ± 0.01^a^	2.00 ± 0.02^c–e^	0.99 ± 0.60^a^	13.53 ± 0.09^b^
AM	84.54 ± 0.05^a^	5.37 ± 0.12^de^	1.03 ± 0.04^a^	1.88 ± 0.03^f–h^	1.01 ± 0.60^a^	10.74 ± 0.12^e^
PH	85.81 ± 0.10^a^	5.27 ± 0.15^e^	1.04 ± 0.04^a^	1.86 ± 0.04^f–j^	1.02 ± 0.59^a^	8.78 ± 0.11^j^
PR	86.39 ± 0.06^a^	3.77 ± 0.25^i^	0.99 ± 0.04^a^	2.07 ± 0.04^ab^	1.01 ± 0.59^a^	11.91 ± 0.18^c^
PM	88.32 ± 0.08^a^	5.84 ± 0.03^a–c^	1.04 ± 0.05^a^	1.83 ± 0.01^h–j^	1.00 ± 0.60^a^	9.45 ± 0.11^h^
Cycle 4						
AH	88.34 ± 0.09^a^	4.77 ± 0.21^f^	0.99 ± 0.08^a^	1.91 ± 0.03^f^	1.00 ± 0.60^a^	10.65 ± 0.07^e^
AR	84.14 ± 0.15^a^	3.70 ± 0.17^i^	1.00 ± 0.02^a^	2.03 ± 0.04^b–d^	0.99 ± 0.59^a^	15.56 ± 0.12^a^
AM	83.00 ± 0.16^a^	4.87 ± 0.02^f^	1.89 ± 0.02^a^	1.89 ± 0.04^fg^	0.98 ± 0.59^a^	11.77 ± 0.04^c^
PH	84.89 ± 0.08^a^	4.87 ± 0.21^f^	1.01 ± 0.02^a^	1.88 ± 0.05^f–i^	1.00 ± 0.59^a^	9.18 ± 0.13^i^
PR	85.64 ± 0.06^a^	3.63 ± 0.15^i^	0.99 ± 0.01^a^	2.09 ± 0.03^a^	0.97 ± 0.60^a^	13.53 ± 0.06^b^
PM	88.18 ± 0.05^a^	5.78 ± 0.03^a–c^	1.04 ± 0.04^a^	1.83 ± 0.01^h–j^	1.03 ± 0.61^a^	10.09 ± 0.04^f^

Values are means of three replicates. Mean values having different superscripts within a column are significantly different (*P* < 0.05). PH, packaged in plastics but thawed with hot water; AH, packaged in aluminum but thawed with hot water; PM, packaged in plastics but thawed with microwave; AM, packaged in aluminum but thawed with microwave; AR, packaged in aluminum but thawed at room temperature; PR, packaged in plastics but thawed at room temperature.

**Table 3:** Effect of freezing and thawing conditions on proximate composition of *Ila* soups

Samples	%Moisture	%Protein	%Fat	%Ash	%Crude fiber	%Carbohydrate
Cycle 0						
I	77.25 ± 0.35^a^	15.94 ± 0.08^a^	2.13 ± 0.04^a^	1.90 ± 0.14^l^	1.15 ± 0.07^a^	1.64 ± 0.60^p^
Cycle 1						
AH	77.32 ± 0.11^a^	14.15 ± 0.17^d–f^	2.08 ± 0.01^a–e^	2.03 ± 0.04^h–j^	1.12 ± 0.01^a–c^	3.23 ± 0.33^n^
AR	75.48 ± 0.20^fg^	13.96 ± 0.06^ef^	2.09 ± 0.03^ab^	2.05 ± 0.02^g–i^	1.12 ± 0.01^a–c^	5.30 ± 0.23^h–j^
AM	76.26 ± 0.25^cd^	14.86 ± 0.42^c^	2.06 ± 0.03^a–f^	2.03 ± 0.02^h–j^	1.11 ± 0.01^a–c^	3.68 ± 0.64^mn^
PH	76.55 ± 0.22^c^	14.55 ± 0.39^cd^	2.08 ± 0.06^a–c^	1.94 ± 0.01^j–l^	1.13 ± 0.01^ab^	3.74 ± 0.34^mn^
PR	76.60 ± 0.40^c^	14.67 ± 0.11^c^	2.10 ± 0.01^a^	2.03 ± 0.07^h–j^	1.11 ± 0.01^a–c^	3.49 ± 0.26^mn^
PM	77.15 ± 0.13^a^	15.44 ± 0.40^b^	2.08 ± 0.03^a–d^	1.92 ± 0.04^kl^	1.14 ± 0.01^ab^	2.28 ± 0.49^o^
Cycle 2						
AH	76.74 ± 0.21^b^	14.04 ± 0.07^ef^	2.02 ± 0.02^b–g^	2.08 ± 0.04^f–i^	1.12 ± 0.02^a–c^	4.00 ± 0.26^lm^
AR	74.24 ± 0.09^i^	14.00 ± 0.04^ef^	2.01 ± 0.01^b–g^	2.11 ± 0.02^e–h^	1.11 ± 0.01^a–c^	6.53 ± 0.09^d–f^
AM	75.58 ± 0.08^fg^	14.10 ± 0.13^d–f^	2.01 ± 0.02^b–g^	2.10 ± 0.01^e–h^	1.11 ± 0.01^a–c^	5.10 ± 0.13^jk^
PH	75.96 ± 0.14^de^	13.82 ± 0.71^f^	1.99 ± 0.01^f–h^	2.13 ± 0.03^e–h^	1.13 ± 0.03^ab^	4.99 ± 0.67^i–k^
PR	75.78 ± 0.29^ef^	13.77 ± 0.21^f^	2.02 ± 0.02^b–g^	2.14 ± 0.06^d–g^	1.05 ± 0.13^c^	5.25 ± 0.51^ij^
PM	76.75 ± 0.18^b^	14.45 ± 0.13^c–e^	2.00 ± 0.02^d–g^	2.00 ± 0.11^i–k^	1.13 ± 0.01^ab^	3.67 ± 0.21^mn^
Cycle 3						
AH	76.62 ± 0.11^bc^	13.71 ± 0.17^f^	2.01 ± 0.01^c–g^	2.11 ± 0.06^e–h^	1.12 ± 0.01^a–c^	4.44 ± 0.12^kl^
AR	72.53 ± 0.10^j^	12.63 ± 0.33^g–i^	1.99 ± 0.01^fg^	2.18 ± 0.02^b–e^	1.08 ± 0.07^a–c^	9.58 ± 0.68^b^
AM	74.90 ± 0.10^h^	13.04 ± 0.61^g^	1.99 ± 0.01^fg^	2.19 ± 0.03^b–e^	1.11 ± 0.01^a–c^	6.76 ± 0.23^d^
PH	75.56 ± 0.13^fg^	13.10 ± 0.19^g^	2.00 ± 0.01^c–g^	2.23 ± 0.01^b–d^	1.12 ± 0.01^a–c^	5.99 ± 0.67^e–g^
PR	75.41 ± 0.23^g^	13.07 ± 0.46^g^	2.00 ± 0.01^e–g^	2.48 ± 0.07^a^	1.13 ± 0.01^ab^	5.91 ± 0.67^f–h^
PM	76.64 ± 0.11^b^	13.65 ± 0.12^f^	2.01 ± 0.01^c–g^	2.11 ± 0.02^e–h^	1.11 ± 0.02^a–c^	4.48 ± 0.09^j–l^
Cycle 4						
AH	76.46 ± 0.12^c^	12.67 ± 0.20^g–i^	1.82 ± 0.07^k^	2.17 ± 0.07^c–f^	1.11 ± 0.01^a–c^	5.77 ± 0.09^g–i^
AR	70.87 ± 0.29^k^	12.26 ± 0.12^ij^	1.84 ± 0.05^jk^	2.19 ± 0.01^b–e^	1.11 ± 0.01^a–c^	11.72 ± 0.21^a^
AM	74.11 ± 0.11^i^	12.41 ± 0.12^hi^	1.84 ± 0.06^jk^	2.24 ± 0.05^b^	1.11 ± 0.01^a–c^	8.30 ± 0.07^c^
PH	75.33 ± 0.08^g^	12.84 ± 0.12^gh^	1.90 ± 0.12^ij^	2.27 ± 0.02^bc^	1.07 ± 0.06^bc^	6.62 ± 0.14^de^
PR	74.67 ± 0.07^h^	11.92 ± 0.07^j^	1.91 ± 0.09^h–j^	2.52 ± 0.06^a^	1.10 ± 0.06^a–c^	7.84 ± 0.05^c^
PM	76.51 ± 0.21^c^	13.86 ± 0.07^f^	1.96 ± 0.06^g–i^	2.17 ± 0.03^c–f^	1.13 ± 0.01^ab^	6.62 ± 0.14^de^

Values are means of three replicates. Mean values having different superscripts within a column are significantly different (*P* < 0.05). PH, packaged in plastics but thawed with hot water; AH, packaged in aluminum but thawed with hot water; PM, packaged in plastics but thawed with microwave; AM, packaged in aluminum but thawed with microwave; AR, packaged in aluminum but thawed at room temperature; PR, packaged in plastics but thawed at room temperature.

**Table 4:** Effect of freezing and thawing conditions on proximate composition of *Ogbono* soups

Samples	%Moisture	%Protein	%Fat	%Ash	%Crude fiber	%Carbohydrate
Cycle 0						
O	68.70 ± 0.14^a^	18.70 ± 0.42^a^	6.12 ± 0.11^a^	4.55 ± 0.21^h^	1.04 ± 0.60^a^	0.89 ± 0.64^n^
Cycle 1						
AH	68.39 ± 0.03^a–e^	18.32 ± 0.20^a–e^	6.06 ± 0.97^ab^	4.75 ± 0.16^c–h^	1.02 ± 0.60^a^	1.45 ± 0.70^k–n^
AR	67.40 ± 0.02^gh^	17.46 ± 0.11^gh^	6.00 ± 0.05^a–d^	4.98 ± 0.11^a–e^	0.68 ± 0.31^a^	3.49 ± 0.47^f–h^
AM	68.11 ± 0.03^b–e^	18.27 ± 0.18^b–e^	6.03 ± 0.11^a–d^	4.82 ± 0.15^b–g^	1.01 ± 0.60^a^	1.75 ± 0.71^k–n^
PH	68.50 ± 0.02^a–d^	18.40 ± 0.19^a–d^	6.08 ± 0.92^a^	4.68 ± 0.16^f–h^	1.03 ± 0.61^a^	1.31 ± 0.73^l–n^
PR	68.11 ± 0.02^h^	17.36 ± 0.07^h^	5.88 ± 0.16^b–f^	4.92 ± 0.16^a–f^	0.67 ± 0.32^a^	3.05 ± 0.39^g–J^
PM	68.54 ± 0.05^ab^	18.48 ± 0.41^ab^	6.09 ± 0.09^a^	4.63 ± 0.18^gh^	1.03 ± 0.60^a^	1.23 ± 0.55^mn^
Cycle 2						
AH	68.08 ± 0.06^b–e^	18.17 ± 0.22^b–e^	5.97 ± 0.09^a–e^	4.83 ± 0.16^b–g^	1.00 ± 0.59^a^	1.95 ± 0.76^i–n^
AR	66.10 ± 0.04^hi^	17.17 ± 0.16^hi^	5.75 ± 0.16^fg^	5.00 ± 0.11^a–d^	0.99 ± 0.58^a^	4.98 ± 0.58^de^
AM	67.53 ± 0.08^c–f^	18.07 ± 0.30^c–f^	5.85 ± 0.17^d–g^	4.88 ± 0.15^a–g^	1.00 ± 0.59^a^	2.67 ± 0.75^h–k^
PH	68.33 ± 0.03^b–e^	18.22 ± 0.^22b–e^	6.04 ± 0.09^a–c^	4.73 ± 0.14^e–h^	1.01 ± 0.59^a^	1.66 ± 0.77^k–n^
PR	67.53 ± 0.03^ij^	16.97 ± 0.03^hi^	5.57 ± 0.07^h^	5.01 ± 0.07^a–c^	0.98 ± 0.59^a^	3.94 ± 0.71^e–g^
PM	68.42 ± 0.04^a–c^	18.46 ± 0.40^a–c^	6.04 ± 0.10^a–c^	4.66 ± 0.15^gh^	1.02 ± 0.61^a^	1.41 ± 0.58^k–n^
Cycle 3						
AH	67.82 ± 0.04^ef^	17.98 ± 0.06^ef^	5.87 ± 0.03^c–f^	4.92 ± 0.13^a–f^	0.98 ± 0.59^a^	2.45 ± 0.74^h–m^
AR	64.80 ± 0.04^jk^	16.68 ± 0.08^ij^	5.54 ± 0.05^h^	5.08 ± 0.06^ab^	0.96 ± 0.59^a^	6.94 ± 0.58^b^
AM	67.00 ± 0.05^fg^	17.78 ± 0.21^fg^	5.79 ± 0.03^e–g^	4.98 ± 0.10^a–f^	0.98 ± 0.59^a^	3.47 ± 0.66^f–h^
PH	68.16 ± 0.04^d–f^	18.04 ± 0.06^d–f^	5.95 ± 0.09^a–e^	4.78 ± 0.15^c–h^	0.99 ± 0.60^a^	2.09 ± 0.81^i–n^
PR	66.95 ± 0.05^kl^	16.45 ± 0.05^jk^	5.31 ± 0.07^ij^	5.06 ± 0.10^ab^	0.95 ± 0.59^a^	5.29 ± 0.58^cd^
PM	68.31 ± 0.03^a–d^	18.40 ± 0.35^a–d^	6.00 ± 0.10^a–d^	4.74 ± 016^d–h^	1.01 ± 0.60^a^	1.54 ± 0.58^k–n^
Cycle 4						
AH	67.54 ± 0.04^gh^	17.55 ± 0.05^gh^	5.74 ± 0.09^fg^	5.00 ± 0.10^a–d^	0.97 ± 0.60^a^	3.20 ± 0.71^f–i^
AR	63.55 ± 0.03^kl^	16.50 ± 0.10^jk^	5.37 ± 0.03^i^	5.14 ± 0.09^a^	0.95 ± 0.60^a^	8.49 ± 0.63^a^
AM	66.45 ± 0.05^gh^	17.43 ± 0.08^gh^	5.68 ± 0.09^gh^	5.07 ± 0.07^ab^	0.96 ± 0.60^a^	4.41 ± 0.72^d–f^
PH	67.96 ± 0.05^fg^	17.75 ± 0.05^fg^	5.87 ± 0.15^c–f^	4.85 ± 0.15^b–g^	0.98 ± 0.60^a^	2.59 ± 0.97^h–l^
PR	66.39 ± 0.04^l^	16.15 ± 0.14^k^	5.15 ± 0.03^j^	5.14 ± 0.05^a^	0.92 ± 0.60^a^	6.24 ± 0.65^bc^
PM	68.20 ± 0.02^b–e^	18.28 ± 0.03^b–e^	5.95 ± 0.13^a–e^	4.76 ± 0.14^c–h^	1.00 ± 0.59^a^	1.81 ± 0.65^j–n^

Values are means of three replicates. Mean values having different superscripts within a column are significantly different (*P* < 0.05). PH, packaged in plastics but thawed with hot water; AH, packaged in aluminum but thawed with hot water; PM, packaged in plastics but thawed with microwave; AM, packaged in aluminum but thawed with microwave; AR, packaged in aluminum but thawed at room temperature; PR, packaged in plastics but thawed at room temperature.

**Table 5:** Effect of freezing and thawing conditions on proximate composition of *Kuka* soups

Samples	%Moisture	%Protein	%Fat	%Ash	%Crude fiber	%Carbohydrate
Cycle 0						
K	78.54 ± 0.06^a^	8.80 ± 0.14^a^	2.29 ± 0.01^a^	2.09 ± 0.01^j^	0.88 ± 0.02^a^	7.41 ± 0.20^n^
Cycle 1						
AH	77.80 ± 0.03^c^	8.70 ± 0.07^a–c^	2.25 ± 0.01^bc^	2.13 ± 0.01^f–h^	0.86 ± 0.01^bc^	8.25 ± 0.06^kl^
AR	76.35 ± 0.48^g^	8.66 ± 0.07^a–d^	2.23 ± 0.01^c–g^	2.15 ± 0.01^c–g^	0.83 ± 0.01^f–i^	9.78 ± 0.42^g^
AM	77.50 ± 0.05^d^	8.67 ± 0.06^a–c^	2.24 ± 0.01^b–e^	2.14 ± 0.01^dh^	0.84 ± 0.01^c–f^	8.61 ± 0.07^j^
PH	78.17 ± 0.04^b^	8.74 ± 1.00^ab^	2.27 ± 0.01^ab^	2.12 ± 0.01^h–j^	0.87 ± 0.01^ab^	7.85 ± 0.12^m^
PR	77.12 ± 0.02^e^	8.64 ± 0.08^a–d^	2.19 ± 0.06^i–k^	2.17 ± 0.01^b–d^	0.82 ± 0.01^h–j^	9.06 ± 0.03^i^
PM	77.90 ± 0.04^c^	8.74 ± 0.13^ab^	2.28 ± 0.01^a^	2.10 ± 0.01^ij^	0.88 ± 0.01^a^	8.10 ± 0.10^l^
Cycle 2						
AH	77.08 ± 0.04^e^	8.64 ± 0.13^a–d^	2.25 ± 0.02^b–e^	2.13 ± 0.02^f–h^	0.85 ± 0.01^c–e^	9.05 ± 0.12^i^
AR	74.72 ± 0.04^k^	8.59 ± 0.12^a–d^	2.21 ± 0.01^g–j^	2.16 ± 0.01^b–e^	0.83 ± 0.01^g–i^	11.49 ± 0.09^d^
AM	76.50 ± 0.05^g^	8.62 ± 0.12^a–d^	2.22 ± 0.10^e–h^	2.15 ± 0.02^d–h^	0.84 ± 0.01^d–g^	9.68 ± 0.12^g^
PH	77.78 ± 0.03^c^	8.66 ± 0.13^a–d^	2.24 ± 0.01^b–e^	2.13 ± 0.01^f–h^	0.85 ± 0.01^c–e^	8.33 ± 0.10^k^
PR	75.71 ± 0.03^i^	8.60 ± 0.12^a–d^	2.19 ± 0.01^h–k^	2.18 ± 0.01^ab^	0.81 ± 0.01^j^`	10.51 ± 0.10^e^
PM	77.31 ± 0.03^d^	8.67 ± 0.13^a–c^	2.27 ± 0.01^ab^	2.12 ± 0.15^h–j^	0.87 ± 0.01^ab^	8.76 ± 0.09^j^
Cycle 3						
AH	76.38 ± 0.03^g^	8.58 ± 0.14^a–d^	2.23 ± 0.01^c–g^	2.14 ± 0.15^d–h^	0.84 ± 0.01^c–f^	9.83 ± 0.12^g^
AR	72.75 ± 0.15^m^	8.54 ± 0.15^b–d^	2.18 ± 0.01^jk^	2.18 ± 0.15^a–c^	0.82 ± 0.01^ij^	13.53 ± 0.09^b^
AM	75.50 ± 0.03^j^	8.56 ± 0.14^b–d^	2.21 ± 0.01^g–j^	2.16 ± 0.03^b–f^	0.83 ± 0.01^e–h^	10.74 ± 0.12^e^
PH	77.38 ± 0.09^d^	8.61 ± 0.16^a–d^	2.24 ± 0.01^c–f^	2.14 ± 0.01^d–h^	0.85 ± 0.01^c–e^	8.78 ± 0.11^j^
PR	74.37 ± 0.04^l^	8.54 ± 0.14^b–d^	2.17 ± 0.01^kl^	2.20 ± 0.02^a^	0.81 ± 0.01^j^	11.91 ± 0.18^c^
PM	76.70 ± 0.05^f^	8.61 ± 0.14^a–d^	2.25 ± 0.01^b–d^	2.13 ± 0.01^e–h^	0.86 ± 0.01^ab^	9.45 ± 0.11^h^
Cycle 4						
AH	75.63 ± 0.07^ij^	8.53 ± 0.08^b–d^	2.21 ± 0.01^g–j^	2.15 ± 0.01^d–h^	0.83 ± 0.01^f–i^	10.65 ± 0.07^e^
AR	70.82 ± 0.15^n^	8.45 ± 0.07^d^	2.18 ± 0.01^kl^	2.19 ± 0.01^ab^	0.81 ± 0.01^j^	15.56 ± 0.12^a^
AM	74.56 ± 0.03^k^	8.49 ± 0.09^cd^	2.19 ± 0.01^h–k^	2.17 ± 0.02^b–d^	0.82 ± 0.01^h–j^	11.77 ± 0.04^c^
PH	77.07 ± 0.03^e^	8.55 ± 0.09^b–d^	2.22 ± 0.01^d–g^	2.14 ± 0.01^d–h^	0.84 ± 0.01^e–h^	9.18 ± 0.13^i^
PR	72.91 ± 0.04^m^	8.44 ± 0.08^d^	2.15 ± 0.01^l^	2.18 ± 0.05^a–c^	0.79 ± 0.01^k^	13.53 ± 0.06^b^
PM	76.17 ± 0.04^h^	8.53 ± 0.05^b–d^	2.24 ± 0.01^b–f^	2.12 ± 0.06^h–j^	0.85 ± 0.06^c–e^	10.09 ± 0.04^f^

Values are means of three replicates. Mean values having different superscripts within a column are significantly different (*P* < 0.05). PH, packaged in plastics but thawed with hot water; AH, packaged in aluminum but thawed with hot water; PM, packaged in plastics but thawed with microwave; AM, packaged in aluminum but thawed with microwave; AR, packaged in aluminum but thawed at room temperature; PR, packaged in plastics but thawed at room temperature.

#### Moisture

The moisture contents obtained for both freshly prepared and selected soup samples subjected to freeze–thaw cycles under different freezing and thawing conditions are presented in Tables 2, 3, 4, 5. The freshly prepared soups had the moisture value of 68.70 ± 0.14% (*Ogbono*), 88.60 ± 0.14% (*Ewedu*), 77.25 ± 0.35% (*Ila*), and 78.54 ± 0.06% (*Kuka*). While the moisture values of the selected soup samples subjected to different frozen and thawing conditions ranged from 63.55 ± 0.03% to 68.54 ± 0.05% (*Ogbono*), 83.00 ± 0.16% to 88.48 ± 0.03% (*Ewedu*), 70.87 ± 0.29% to 77.15 ± 0.13% (*Ila*), and 70.82 ± 0.04% to 77.90 ± 0.04% (*Kuka*). The moisture content of the soups determines their susceptibility to microbial attack and hence spoilage (Olusanya 2008). The moisture content of freshly prepared “Ogbono” (68.70 ± 0.14%) was lower than those of freshly prepared “Ila,” “Ewedu,”and “Kuka.” This indicated that freshly prepared *Ogbono* with lower moisture content might have storage advantage over others. Soups that were packaged in plastic and aluminum containers and thawed at 5 days interval for four freeze–thaw cycles at room temperature had the lowest moisture content retention. While soups that were packaged in plastic and thawed in microwave oven had the highest moisture content retention when compared with the freshly prepared soup (Tables 2, 3, 4, 5). This might be as a result of fast thawing rate exhibited by microwave‐thawed plastic soups which prevented weight loss during thawing. An increase in the number of freeze–thaw cycles (1–4) resulted in the considerable amount of loss in the moisture content (Tables 2, 3, 4, 5) of soups subjected to freezing and thawing conditions. Repeated melting during thawing and reformation of ice crystals during freezing in multiple freeze–thaw situations was clearly detrimental to food quality, by causing mechanical damage to cell membrane and loss of water‐holding capacity of food (Boonsumrej et al. 2007). Frozen foods are not homogenous in nature since they always contain frozen and unfrozen phase and a non‐uniform distribution of food components. These components differ greatly in their ability to absorb radiofrequency energy and this tends to cause localized area to overheat before other areas are thawed (Boonsumrej et al. 2007). Therefore, this resulted into considerable moisture loss in all the soup samples packaged in both plastic and aluminum containers and thawed in microwave oven at every freeze–thaw cycle. Plastic containers absorbed heat and retained it to thaw the soups packed in them at a considerable rate in hot water and microwave‐thawing conditions, while aluminum containers conducted heat away from the soups they contained, creating less effective thawing rate in hot water and microwave‐thawing conditions, this might be due to high heat transfer coefficient of the aluminum containers (Singh and Heldman 2000). Although microwave thawing produced rapid thawing in plastic containers, and much more uniform than heating by conduction (Karel and Lund 2003), little amount of moisture was lost compared to other conditions stated earlier where considerable losses were observed.

#### Protein

Tables 2, 3, 4, 5 show the protein content obtained for both freshly prepared and selected soup samples subjected to freeze–thaw cycles under different freezing and thawing conditions. The freshly prepared soups had protein values of 18.70 ± 0.42% (*Ogbono*), 6.00 ± 0.01% (*Ewedu*), 15.94 ± 0.08% (*Ila*), and 8.80 ± 0.14% (*Kuka*). While the protein values of the selected soup samples subjected to different frozen and thawing conditions ranged from 16.15 ± 0.14% to 18.70 ± 0.42% (*Ogbono*), 3.63 ± 0.15% to 5.92 ± 0.07% (*Ewedu*), 11.92 ± 0.07% to 15.44 ± 0.40% (*Ila*), and 8.44 ± 0.08% to 8.74 ± 0.13% (*Kuka*).

Proteins are important in the body due to their numerous roles (Uwakwe and Ayalogu 1998; DuruMajesty et al. 2012). Of the four soups under investigation, freshly prepared *Ogbono* had the highest protein content. Freshly prepared *Ewedu* and *Kuka* with lower protein contents might not be able to contribute significantly to the daily protein requirements of 22–56 g (NRC 1975). During freezing and thawing of soups, it was observed that soups that were packaged in plastic and aluminum containers and thawed at 5 days interval for four freeze–thaw cycles at room temperature had the lowest protein content retention (Tables 2, 3, 4, 5). While soups that were packaged in plastic and thawed in microwave oven had the highest protein content retention when compared with the freshly prepared soup (Tables 2, 3, 4, 5). Considerable amount of losses in the protein contents of soups subjected to freezing and thawing conditions were observed as the freeze–thaw cycles (1–4) increased. Protein denaturation can be defined as functionality caused by changes in the protein structure due to the disruption of chemical bonds and by secondary interactions with other constituents (Alizadeh et al. 2009). The reduction in crude protein of the soups during ice storage could be attributed to the gradual degradation of the initial crude protein to more volatile products as total volatile bases (TVB), trimethyl amine (TMA) hydrogen sulfide, and ammonia and the changes in protein and lipid content might be associated with the leaching out to ice of some of the lipid fractions (Obemeata and Christopher 2012). The reduction in crude protein content of the frozen soups might also have been due to a decrease in salt‐soluble protein and water‐soluble protein (Chomnawang et al. 2007) or due to autolytic deterioration associated with the actions of endogenous enzymes and bacteria (Hultman and Rustard 2004). Losses in protein during thawing might be due to heat disruption (Alizadeh et al. 2007b) (hot water thawing), energy disruption (microwave thawing) (Boonsumrej et al. 2007), and microbial activities (room temperature thawing) (Leygonie et al. 2012).

#### Fat

Presented in Tables 2, 3, 4, 5 are the fat content obtained for both freshly prepared and selected soup samples subjected to freeze–thaw cycles under different freezing and thawing conditions. The freshly prepared soups had the fat value of 6.12 ± 0.11% (*Ogbono*), 1.05 ± 0.05% (*Ewedu*), 2.13 ± 0.04% (*Ila*), and 2.29 ± 0.01% (*Kuka*). While the fat values of the selected soup samples subjected to different frozen and thawing conditions ranged from 5.15 ± 0.03% to 6.12 ± 0.11% (*Ogbono*), 1.00 ± 0.02% to 1.05 ± 0.05% (*Ewedu*), 1.82 ± 0.07% to 2.08 ± 0.03% (*Ila*), and 2.15 ± 0.01% to 2.28 ± 0.01% (*Kuka*).

However, soups packaged in plastic and aluminum containers and thawed at 5 days interval for four freeze–thaw cycles at room temperature had the lowest fat retention (Tables 2, 3, 4, 5). While soups that were packaged in plastic and thawed in microwave oven had the highest fat content retention when compared with the freshly prepared soup (Tables 2, 3, 4, 5). Fats are saturated lipids at room temperature (Kritchevsky 1996; DuruMajesty et al. 2012) which are known to play protective roles in the body system (Olusanya 2008). Some important fatty acids such as omega‐3‐fatty acid, etc., are derived from fats. These fatty acids are noted for their roles in the body system (Obidoa et al. 2010). The crude fat content of the soups were in the range of 1.05 ± 0.05% to 6.12 ± 0.11%. Decrease in the fat contents of soups subjected to freezing and thawing conditions varies considerably as the freeze–thaw cycle increases (1–4). Variation in the fat contents might be as a result of different quantities of palm oil added to the soups as stated in the recipe book. For all the conditions that the selected soups were subjected to, the fat content reduced during the freeze–thaw cycles. The reduction in fat content indicates an increase in lipid oxidation. This could be due to the release of oxidative enzymes and pro‐oxidants from various rupture cellular organelles (Boonsumrej et al. 2007).

#### Crude fiber

The results of crude fiber content obtained for both freshly prepared and selected soup samples subjected to freeze–thaw cycles under different freezing and thawing conditions are shown in Tables 2, 3, 4, 5. The freshly prepared soups had the crude fiber value of 1.04 ± 0.60% (*Ogbono*), 1.04 ± 0.60% (*Ewedu*), 1.15 ± 0.07% (*Ila*), and 0.88 ± 0.02% (*Kuka*). While the crude fiber values of the selected soup samples subjected to different frozen and thawing conditions ranged from 0.92 ± 0.60% to 1.03 ± 0.60% (*Ogbono*), 1.00 ± 0.59% to 1.03 ± 0.61% (*Ewedu*), 1.07 ± 0.06% to 1.14 ± 0.01% (*Ila*), and 0.79 ± 0.01% to 0.88 ± 0.01% (*Kuka*).

Adequate intake of dietary fiber can lower the level of serum cholesterol and reduce the risk of developing hypertension, constipation, diabetes, colon cancer, and coronary heart disease (Ishida et al. 2000). The fiber content of the freshly prepared soups ranged between 0.875 ± 0.02% and 1.47 ± 0.02%, with *Ila* having the highest fiber content. The lowest crude fiber retention was obtained at every freeze–thaw cycle in soups packaged in plastic and aluminum containers but thawed at room temperature. While soups that were packaged in plastic and thawed in microwave oven had the highest crude fiber content retention when compared with the freshly prepared soup (Tables 2, 3, 4, 5). An increase in the number of freeze–thaw cycles (1–4) resulted to the considerable amount of loss in the crude fiber content of soups subjected to freezing and thawing conditions. Losses in crude fiber from all these soups were most probably dominated by enzyme‐induced degradation. The variation in the percentage loss of crude fiber demonstrated the differences in vulnerabilities of the selected soup to spoilage at every freeze–thaw cycle. This might be due to mechanical stress caused by freezing and thawing, surface area, and their differing enzymatic activities (Martinez‐Romero et al. 2004). Minimal losses in crude fiber of soup samples packaged in plastic and thawed in microwave oven was attributed to the short thawing time which limited enzymatic activities.

#### Ash

Tables 2, 3, 4, 5 present the ash content obtained for both freshly prepared and selected soup samples subjected to freeze–thaw cycles under different freezing and thawing conditions. The freshly prepared soups had the ash value of 4.55 ± 0.21% (*Ogbono*), 1.81 ± 0.01% (*Ewedu*), 1.90 ± 0.14% (*Ila*), and 2.09 ± 0.01% (*Kuka*). While the ash values of the selected soup samples subjected to different frozen and thawing conditions ranged from 4.55 ± 0.11% to 5.14 ± 0.09% (*Ogbono*), 1.81 ± 0.01% to 2.03 ± 0.04% (*Ewedu*), 1.90 ± 0.04% to 2.52 ± 0.06% (*Ila*), and 2.09 ± 0.01% to 2.18 ± 0.05% (*Kuka*). The ash content was moderate in all the soups samples subjected to freezing and thawing conditions. Ash content is an index of mineral contents in biota (Akubugwo et al. 2007). The observed ash content from the freshly prepared soups ranged between 1.81 ± 0.01% and 4.55 ± 0.21%, with freshly prepared *Ogbono* having the highest ash content. This could mean that the minerals in freshly prepared *Ogbono* are higher than that of others. Accordingly, reductions in other chemical components might result into corresponding increase in ash contents due to concentration of soluble solids with relatively chemically stable products.

#### Carbohydrate

Tables 2, 3, 4, 5 show the carbohydrate values obtained for both freshly prepared and selected soup samples subjected to freeze–thaw cycles under different freezing and thawing conditions. The freshly prepared soups had the carbohydrate values of 0.89 ± 0.64% (*Ogbono*), 7.41 ± 0.20% (*Ewedu*), 1.64 ± 0.60% (*Ila*), and 7.41 ± 0.20% (*Kuka*). While the carbohydrate values of the selected soup samples subjected to different frozen and thawing conditions ranged from 1.23 ± 0.55% to 8.49 ± 0.63% (*Ogbono*), 7.85 ± 0.12% to 13.53 ± 0.06% (*Ewedu*), 2.28 ± 0.49% to 11.72 ± 0.21% (*Ila*), and 7.85 ± 0.12% to 15.56 ± 0.12% (*Kuka*).

During freezing and thawing of soups, it was observed that soups that were packaged in plastic and aluminum containers and thawed at 5 days interval for four freeze–thaw cycles at room temperature had the lowest carbohydrate retention when compared with microwave and hot water–thawed soups packaged in plastic containers (Tables 2, 3, 4, 5). While soups that were packaged in plastic and thawed in microwave oven had the lowest carbohydrate content when compared with the freshly prepared soup (Tables 2, 3, 4, 5). An increase in the number of freeze–thaw cycles (1–4) resulted to the considerable increase in the carbohydrate content of soups subjected to freezing and thawing conditions. Increase in carbohydrate content at every freeze–thaw cycle might be as a result of loss of moisture which causes redistribution of chemical composition within the food. The recommended dietary allowance (RDA) values of carbohydrate for adults and pregnant and lactating mothers are 130, 175, and 210 g, respectively (DuruMajesty et al. 2012). The carbohydrate contents of the soups were very low, but this is not a concern since they are been consumed along with starch‐based dietary staples (Kayode et al. 2010).

### Effect of freezing and thawing conditions on mineral composition of some selected soups

The effect of freezing and thawing conditions on the mineral composition of *Ogbono*,* Ewedu*,* Ila*, and *Kuka* soups subjected to frozen storage were presented in Tables 6, 7, 8, 9. There were significant differences (*P* < 0.05) in the mineral composition of *Ogbono*,* Ewedu*,* Ila*, and *Kuka* soups when subjected to the above frozen and thawing conditions as compared to the freshly prepared *Ogbono*,* Ewedu*,* Ila*, and *Kuka* soups.

**Table 6:** Effect of freezing and thawing conditions on mineral composition of *Ogbono* soups

Sample	Iron (mg/100 g)	Calcium (mg/100 g)	Sodium (mg/100 g)	Potassium (mg/100 g)	Phosphorus (mg/100 g)
Cycle 0					
O	6.34 ± 0.10^a^	73.71 ± 0.04^a^	160.21 ± 0.26^a^	260.50 ± 0.50^a^	337.19 ± 0.07^a^
Cycle 1					
AH	6.14 ± 0.03^c–f^	73.45 ± 0.06^c–e^	159.66 ± 0.03^b–d^	259.81 ± 0.11^cd^	336.97 ± 0.07^b–d^
AR	6.11 ± 0.03^e–g^	73.32 ± 0.02^g–i^	159.35 ± 0.10^g–k^	259.63 ± 0.07^d–f^	336.80 ± 0.08^d–g^
AM	6.11 ± 0.01^e–g^	73.34 ± 0.06^f–i^	159.55 ± 0.07^c–f^	259.74 ± 0.08^c–e^	336.86 ± 0.08^d–f^
PH	6.18 ± 0.05^b–d^	73.51 ± 0.04^c^	159.76 ± 0.10^b^	259.93 ± 0.07^bc^	337.08 ± 0.07^a–c^
PR	6.09 ± 0.02^fg^	73.31 ± 0.06^g–i^	159.44 ± 0.10^e–h^	259.63 ± 0.06^d–f^	336.81 ± 0.07^d–g^
PM	6.23 ± 0.10^b^	73.62 ± 0.05^b^	159.93 ± 0.16^a^	260.03 ± 0.05^b^	337.13 ± 0.12^ab^
Cycle 2					
AH	6.10 ± 0.02^e–g^	73.38 ± 0.07^e–h^	159.50 ± 0.09^d–g^	259.46 ± 0.12^f–i^	336.79 ± 0.11^d–h^
AR	6.09 ± 0.01^fg^	73.30 ± 0.07^g–i^	159.40 ± 0.06^e–i^	259.33 ± 0.04^h–j^	336.72 ± 0.08^e–h^
AM	6.09 ± 0.02^fg^	73.32 ± 0.09^g–i^	159.44 ± 0.07^e–h^	259.39 ± 0.07^g–j^	336.73 ± 0.10^e–h^
PH	6.13 ± 0.02^c–g^	73.43 ± 0.10^c–f^	159.56 ± 0.11^c–e^	259.51 ± 0.07^f–h^	336.90 ± 0.10^c–e^
PR	6.08 ± 0.01^fg^	73.29 ± 0.08^g–i^	159.37 ± 0.06^f–j^	259.34 ± 0.04^g–j^	336.72 ± 0.12^e–h^
PM	6.19 ± 0.01^bc^	73.49 ± 0.04^cd^	159.71 ± 0.17^bc^	259.75 ± 0.15^c–e^	337.06 ± 0.09^a–c^
Cycle 3					
AH	6.12 ± 0.10^d–g^	73.38 ± 0.03^e–h^	159.27 ± 0.07^h–k^	259.41 ± 0.04^f–j^	336.66 ± 0.08^f–j^
AR	6.08 ± 0.01^fg^	73.32 ± 0.03^g–i^	159.19 ± 0.07^jk^	259.34 ± 0.03^g–j^	336.29 ± 0.07^l^
AM	6.09 ± 0.02^fg^	73.35 ± 0.03^f–i^	159.23 ± 0.06^i–k^	259.37 ± 0.04^g–j^	336.60 ± 0.08^h–j^
PH	6.14 ± 0.01^c–f^	73.42 ± 0.04^d–f^	159.31 ± 0.09^g–k^	259.43 ± 0.12^f–j^	336.76 ± 0.08^e–h^
PR	6.07 ± 0.02^g^	73.31 ± 0.03^g–i^	159.20 ± 0.06^jk^	259.33 ± 0.03^h–j^	336.33 ± 0.11^kl^
PM	6.16 ± 0.02^c–e^	73.44 ± 0.04^c–e^	159.45 ± 0.09^e–g^	259.57 ± 0.09^e–g^	336.97 ± 0.07^b–d^
Cycle 4					
AH	6.10 ± 0.01^e–g^	73.30 ± 0.02^g–i^	159.27 ± 0.02^h–k^	259.24 ± 0.04^ij^	336.77 ± 0.28^e–h^
AR	6.08 ± 0.00^fg^	73.29 ± 0.01^g–i^	159.18 ± 0.03^k^	259.27 ± 0.05^h–j^	336.49 ± 0.07^jk^
AM	6.09 ± 0.03^fg^	73.29 ± 0.02^g–i^	159.22 ± 0.03^i–k^	259.21 ± 0.04^j^	336.65 ± 0.06^g–j^
PH	6.11 ± 0.01^e–g^	73.34 ± 0.02^f–i^	159.31 ± 0.06^g–k^	259.29 ± 0.04^h–j^	336.69 ± 0.15^f–i^
PR	6.08 ± 0.02^fg^	73.28 ± 0.02^i^	159.21 ± 0.03^jk^	259.26 ± 0.04^ij^	336.50 ± 0.05^i–k^
PM	6.13 ± 0.02^c–g^	73.38 ± 0.02^e–g^	159.37 ± 0.07^f–j^	259.46 ± 0.09^f–i^	336.82 ± 0.07^d–g^

Values are means of three replicates. Mean values having different superscripts within a column are significantly different (*P* < 0.05). PH, packaged in plastics but thawed with hot water; AH, packaged in aluminum but thawed with hot water; PM, packaged in plastics but thawed with microwave; AM, packaged in aluminum but thawed with microwave; AR, packaged in aluminum but thawed at room temperature; PR, packaged in plastics but thawed at room temperature.

**Table 7:** Effect of freezing and thawing conditions on mineral composition of *Ewedu* soups

Samples	Iron (mg/100 g)	Calcium (mg/100 g)	Sodium (mg/100 g)	Potassium (mg/100 g)	Phosphorus (mg/100 g)
Cycle 0					
E	6.75 ± 0.08^a^	147.53 ± 0.25^a^	150.29 ± 0.05^a^	206.30 ± 0.05^a^	312.43 ± 0.08^a^
Cycle 1					
AH	6.65 ± 0.06^bc^	146.30 ± 0.17^c^	149.45 ± 0.12^c–e^	205.75 ± 0.12^c–f^	311.60 ± 0.17^cd^
AR	6.59 ± 0.05^c–e^	144.79 ± 0.10^f^	149.32 ± 0.13^c–f^	205.47 ± 0.08^e–g^	311.27 ± 0.10^f–h^
AM	6.62 ± 0.07^cd^	145.67 ± 0.11^d^	149.37 ± 0.14^c–f^	205.56 ± 0.11^d–g^	311.43 ± 0.21^d–f^
PH	6.71 ± 0.03^ab^	147.12 ± 0.07^b^	149.61 ± 0.23^bc^	205.97 ± 0.17^a–c^	311.89 ± 0.11^b^
PR	6.60 ± 0.02^c–e^	144.18 ± 0.05^h^	149.24 ± 0.10^c–g^	205.41 ± 0.08^f–h^	311.19 ± 0.08^f–i^
PM	6.71 ± 0.07^ab^	147.12 ± 0.24^b^	149.90 ± 0.43^b^	206.16 ± 0.15^ab^	312.29 ± 0.05^a^
Cycle 2					
AH	6.48 ± 0.02^fg^	144.97 ± 0.17^f^	149.14 ± 0.12^e–h^	205.41 ± 0.17^f–h^	311.33 ± 0.12^e–g^
AR	6.56 ± 0.04^d–f^	144.13 ± 0.04^h^	148.71 ± 0.62^h–l^	205.29 ± 0.46^g–i^	311.23 ± 0.13^f–h^
AM	6.43 ± 0.03^gh^	144.68 ± 0.13^fg^	149.09 ± 0.03^e–h^	205.36 ± 0.07^f–h^	311.29 ± 0.13^fg^
PH	6.53 ± 0.02^ef^	145.35 ± 0.11^e^	149.43 ± 0.09^c–e^	205.83 ± 0.29^b–e^	311.77 ± 0.19^bc^
PR	6.54 ± 0.04^ef^	143.19 ± 0.03j	148.63 ± 0.64^h–k^	205.22 ± 0.08^g–i^	311.15 ± 0.13^g–i^
PM	6.65 ± 0.06^bc^	146.34 ± 0.11^c^	149.58 ± 0.26^b–d^	205.93 ± 0.29^a–d^	312.02 ± 0.02^b^
Cycle 3					
AH	6.40 ± 0.02^hi^	144.19 ± 0.08^h^	148.83 ± 0.05^g–k^	205.00 ± 0.10^h–j^	311.36 ± 0.17^d–g^
AR	6.33 ± 0.02^i–l^	143.07 ± 0.50^jk^	148.45 ± 0.06^i–m^	204.80 ± 0.40^j^	311.14 ± 0.09^g–i^
AM	6.36 ± 0.03^h–j^	143.23 ± 0.07^j^	148.57 ± 0.09^h–l^	204.92 ± 0.16^ij^	311.22 ± 0.13^f–h^
PH	6.54 ± 0.36^ef^	144.89 ± 0.22^f^	148.86 ± 0.26^g–j^	205.41 ± 0.23^f–h^	311.55 ± 0.22^c–e^
PR	6.29 ± 0.03^j–l^	142.33 ± 0.14^l^	148.40 ± 0.06^j–m^	204.72 ± 0.37^j^	311.10 ± 0.10^g–j^
PM	6.59 ± 0.04^c–e^	146.25 ± 0.08^c^	149.17 ± 0.07^d–g^	205.48 ± 0.48^j^	311.89 ± 0.10^b^
Cycle 4					
AH	6.34 ± 0.01^i–k^	143.67 ± 0.29^i^	148.30 ± 0.09^k–m^	204.80 ± 0.25	310.94 ± 0.13^ij^
AR	6.26 ± 0.04^lm^	142.48 ± 0.07^l^	148.16 ± 0.07^lm^	204.90 ± 0.20^ij^	311.00 ± 0.12^h–j^
AM	6.28 ± 0.04^k–m^	142.84 ± 0.05^k^	148.18 ± 0.08^lm^	204.74 ± 0.28^j^	310.87 ± 0.27^j^
PH	6.49 ± 0.03^fg^	144.43 ± 0.16^gh^	148.62 ± 0.05^h–k^	205.02 ± 0.07^h–j^	311.27 ± 0.08^f–h^
PR	6.21 ± 0.03^m^	141.51 ± 0.24^m^	148.12 ± 0.08^m^	204.90 ± 0.20^ij^	310.93 ± 0.21^ij^
PM	6.50 ± 0.06^f^	145.68 ± 0.18^d^	148.96 ± 0.2^f–i^	205.47 ± 0.18^e–g^	311.56 ± 0.11^c–e^

Values are means of three replicates. Mean values having different superscripts within a column are significantly different (*P* < 0.05). PH, packaged in plastics but thawed with hot water; AH, packaged in aluminum but thawed with hot water; PM, packaged in plastics but thawed with microwave; AM, packaged in aluminum but thawed with microwave; AR, packaged in aluminum but thawed at room temperature; PR, packaged in plastics but thawed at room temperature.

**Table 8:** Effect of freezing and thawing conditions on mineral composition of *Ila* soups

Sample	Iron (mg/100 g)	Calcium (mg/100 g)	Sodium (mg/100 g)	Potassium (mg/100 g)	Phosphorus (mg/100 g)
Cycle 0					
I	5.30 ± 0.04^a^	73.57 ± 0.18^a^	152.48 ± 1.24^a^	210.24 ± 0.27^a^	238.84 ± 1.15^a^
Cycle 1					
AH	5.27 ± 0.01^a^	72.88 ± 0.34^b^	150.21 ± 0.15^ef^	209.20 ± 0.12^a–c^	234.91 ± 0.79^c–e^
AR	5.24 ± 0.02^a–d^	71.62 ± 0.12^c–e^	148.37 ± 0.07^ij^	206.41 ± 0.95^e–g^	232.72 ± 0.74^gh^
AM	5.24 ± 0.02^a–d^	71.88 ± 0.11^c^	149.01 ± 0.10^hi^	208.57 ± 0.76^b–d^	234.04 ± 0.65^ef^
PH	5.28 ± 0.04^a^	72.49 ± 0.20^b^	151.48 ± 0.34^c^	209.55 ± 0.11^bc^	235.95 ± 0.61^c^
PR	5.14 ± 0.03^ij^	71.48 ± 0.04^c–f^	147.82 ± 0.12^jk^	206.12 ± 0.34^g^	232.00 ± 0.58^h^
PM	5.28 ± 0.02^a^	72.60 ± 0.37^b^	152.24 ± 1.26^ab^	210.04 ± 0.36^a^	237.37 ± 0.88^b^
Cycle 2					
AH	5.26 ± 0.02^a–c^	71.38 ± 0.21^d–f^	149.46 ± 0.91^gh^	207.46 ± 1.00^d–f^	233.36 ± 0.37^fg^
AR	5.22 ± 0.01^de^	70.71 ± 0.32^gh^	147.54 ± 0.31^k^	204.52 ± 0.84^hi^	231.69 ± 0.38^hi^
AM	5.24 ± 0.01^a–d^	70.87 ± 0.40^gh^	148.75 ± 0.09^i^	205.59 ± 0.56^gh^	232.40 ± 0.52^gh^
PH	5.23 ± 0.03^bc^	71.48 ± 0.21^c–f^	151.26 ± 0.07^cd^	208.30 ± 0.22^c–e^	234.71 ± 0.59^de^
PR	5.11 ± 0.01^j^	70.72 ± 0.11^gh^	146.59 ± 0.18^l^	204.53 ± 0.52^hi^	230.67 ± 0.48^ij^
PM	5.26 ± 0.03^bc^	71.80 ± 0.30^cd^	151.71 ± 0.28^bc^	209.57 ± 0.10^bc^	235.72 ± 0.52^cd^
Cycle 3					
AH	5.21 ± 0.01^e–g^	71.04 ± 0.09^fg^	148.76 ± 0.09^i^	206.04 ± 0.34^g^	235.72 ± 0.57^h^
AR	5.15 ± 0.01^hi^	69.85 ± 0.20^jk^	146.21 ± 0.21^lm^	202.93 ± 1.14^ij^	229.91 ± 0.58^jk^
AM	5.18 ± 0.02^f–h^	70.14 ± 0.23^ij^	147.75 ± 0.12^jk^	203.67 ± 0.62^hi^	230.78 ± 0.62^ij^
PH	5.22 ± 0.01^de^	71.16 ± 0.10^fg^	150.05 ± 0.04^fg^	207.26 ± 0.06^d–f^	233.37 ± 0.42^fg^
PR	5.05 ± 0.02^k^	69.05 ± 0.53^n^	145.28 ± 0.07^n^	202.39 ± 0.04^j^	228.89 ± 0.45^kl^
PM	5.23 ± 0.01^b–d^	71.33 ± 0.20^ef^	151.06 ± 0.06^cd^	208.68 ± 0.12^bc^	234.23 ± 0.26^ef^
Cycle 4					
AH	5.16 ± 0.01^hi^	70.43 ± 0.18^hi^	146.79 ± 0.17^l^	204.24 ± 0.75^j^	230.69 ± 0.08^ij^
AR	5.15 ± 0.02^h–j^	69.22 ± 0.14^lm^	145.67 ± 0.21^mn^	201.06 ± 0.74^k^	228.97 ± 0.46^kl^
AM	5.16 ± 0.02^hi^	69.49 ± 0.11^kl^	146.67 ± 0.20^l^	202.66 ± 0.61^ij^	229.66 ± 0.50^jk^
PH	5.18 ± 0.02^g–i^	70.51 ± 0.21^hi^	149.66 ± 0.09^f–h^	206.30 ± 0.12^fg^	231.56 ± 0.78^hi^
PR	4.97 ± 0.03^l^	68.11 ± 0.32^m^	144.39 ± 0.14^o^	200.38 ± 0.97^k^	227.99 ± 0.30^l^
PM	5.22 ± 0.03^ef^	70.74 ± 0.23^gh^	150.73 ± 0.28^de^	208.36 ± 0.58^c–e^	233.41 ± 0.73^fg^

Values are means of three replicates. Mean values having different superscripts within a column are significantly different (*P* < 0.05). PH, packaged in plastics but thawed with hot water; AH, packaged in aluminum but thawed with hot water; PM, packaged in plastics but thawed with microwave; AM, packaged in aluminum but thawed with microwave; AR, packaged in aluminum but thawed at room temperature; PR, packaged in plastics but thawed at room temperature.

**Table 9:** Effect of freezing and thawing conditions on mineral composition of *Kuka* soups

Samples	Iron (mg/100 g)	Calcium (mg/100 g)	Sodium (mg/100 g)	Potassium (mg/100 g)	Phosphorus (mg/100 g)
Cycle 0					
K	6.28 ± 0.02^a^	190.83 ± 0.08^a^	231.72 ± 0.03^b^	302.07 ± 0.06^a^	323.52 ± 0.50^a^
Cycle 1					
AH	6.23 ± 0.03^bc^	190.66 ± 0.05^a–f^	231.56 ± 0.07^b^	301.82 ± 0.01^b–d^	323.17 ± 0.01^a–c^
AR	6.19 ± 0.03^e–g^	190.47 ± 0.10^d–i^	231.38 ± 0.10^b^	301.74 ± 0.03^c–f^	323.11 ± 0.01^a–d^
AM	6.21 ± 0.02^c–f^	190.59 ± 0.13^a–h^	261.44 ± 0.09^a^	301.80 ± 0.32^c–e^	323.14 ± 0.02^a–c^
PH	6.23 ± 0.02^bc^	190.76 ± 0.10^a–c^	231.59 ± 0.05^b^	301.88 ± 0.02^bc^	323.21 ± 0.03^a–c^
PR	6.18 ± 0.02^f–h^	190.41 ± 0.03^f–i^	261.19 ± 0.05^a^	301.51 ± 0.04^g–i^	323.09 ± 0.01^a–d^
PM	6.24 ± 0.02^b^	190.79 ± 0.10^ab^	231.64 ± 0.07^b^	301.97 ± 0.07^ab^	323.37 ± 0.07^ab^
Cycle 2					
AH	6.21 ± 0.01^b–e^	190.71 ± 0.11^a–d^	231.34 ± 0.11^b^	301.63 ± 0.06^e–g^	322.89 ± 0.11^c–e^
AR	6.19 ± 0.01^d–g^	190.44 ± 0.12^e–i^	231.38 ± 0.16^b^	301.58 ± 0.06^f–h^	322.66 ± 0.20^d–g^
AM	6.21 ± 0.01^b–f^	190.61 ± 0.16^a–g^	231.31 ± 0.08^b^	301.59 ± 0.13^f–h^	322.81 ± 0.12^c–f^
PH	6.22 ± 0.01^b–d^	190.59 ± 0.03^a–h^	231.49 ± 0.07^b^	301.82 ± 0.07^b–d^	323.06 ± 0.04^b–d^
PR	6.15 ± 0.02^hi^	190.37 ± 0.07^g–i^	231.16 ± 0.03^b^	301.40 ± 0.03^ij^	322.55 ± 0.12^e–g^
PM	6.23 ± 0.01^bc^	190.67 ± 0.05^a–e^	231.56 ± 0.06^b^	301.88 ± 0.08^bc^	323.10 ± 0.02^a–d^
Cycle 3					
AH	6.20 ± 0.10^c–f^	190.52 ± 0.02^c–i^	231.43 ± 0.08^b^	301.75 ± 0.07^c–f^	322.51 ± 0.04^e–g^
AR	6.11 ± 0.02^jk^	190.33 ± 0.23^i^	231.48 ± 0.11^b^	301.45 ± 0.22^h–j^	322.26 ± 0.17^g–i^
AM	6.19 ± 0.01^e–g^	190.33 ± 0.21^hi^	231.35 ± 0.06^b^	301.50 ± 0.23^g–i^	322.38 ± 0.27^f–h^
PH	6.21 ± 0.01^b–f^	190.50 ± 0.09^c–i^	231.47 ± 0.05^b^	301.72 ± 0.03^c–f^	322.67 ± 0.29^d–g^
PR	6.10 ± 0.02^k^	190.08 ± 0.13^j^	231.13 ± 0.02^b^	301.32 ± 0.03^j^	322.25 ± 0.15^g–i^
PM	6.22 ± 0.01^b–e^	190.56 ± 0.11^b–i^	231.48 ± 0.06^b^	301.80 ± 0.05^c–e^	322.89 ± 0.21^c–e^
Cycle 4					
AH	6.18 ± 0.01^f–h^	190.50 ± 0.10^c–i^	231.36 ± 0.06^b^	301.65 ± 0.10^e–g^	321.94 ± 0.24^ij^
AR	6.14 ± 0.03^ij^	190.31 ± 0.03^i^	231.40 ± 0.07^b^	301.58 ± 0.06^f–h^	321.85 ± 0.17^ij^
AM	6.16 ± 0.02^g–i^	190.36 ± 0.05^g–i^	231.31 ± 0.05^b^	301.58 ± 0.07^f–h^	321.97 ± 0.09^h–j^
PH	6.19 ± 0.01^e–g^	190.46 ± 0.10^d–i^	231.39 ± 0.06^b^	301.70 ± 0.07^d–f^	322.86 ± .0.55^c–e^
PR	6.10 ± 0.02^k^	189.60 ± 0.39^k^	230.83 ± 0.06^b^	301.16 ± 0.13^k^	321.76 ± 0.13^j^
PM	6.20 ± 0.01^c–f^	190.50 ± 0.13^c–i^	231.43 ± 0.08^b^	301.74 ± 0.06^c–f^	322.91 ± 0.51^c–e^

Values are means of three replicates. Mean values having different superscripts within a column are significantly different (*P* < 0.05). PH, packaged in plastics but thawed with hot water; AH, packaged in aluminum but thawed with hot water; PM, packaged in plastics but thawed with microwave; AM, packaged in aluminum but thawed with microwave; AR, packaged in aluminum but thawed at room temperature; PR, packaged in plastics but thawed at room temperature.

#### Iron

The freshly prepared soups had the iron values of 6.34 ± 0.10 mg/100 g (*Ogbono*), 6.75 ± 0.08 mg/100 g (*Ewedu*), 5.30 ± 0.04 mg/100 g (*Ila*), and 6.28 ± 0.02 mg/100 g (*Kuka*). The range of iron contents of the selected soup samples subjected to different frozen and thawing conditions were as follows: 6.08 ± 0.00–6.23 ± 0.10 mg/100 g (*Ogbono*), 6.21 ± 0.03–6.71 ± 0.07 mg/100 g (*Ewedu*), 4.97 ± 0.03–5.28 ± 0.02 mg/100 g (*Ila*), and 6.16 ± 0.02–6.24 ± 0.02 mg/100 g (*Kuka*). Freshly prepared *Ewedu* was observed to have the highest concentration of iron, with freshly prepared *Ila* having the lowest concentration.

Different minerals perform important body functions including oxygen transport, nerve‐muscle function, enzyme activity, energy metabolism, and formation of some hormones, water balance, acid–base balance, and growth tissues (Hegarty 1995; Sanni et al. 2010). Inadequate mineral intake may become a problem, most especially for the vulnerable groups such as the infants and young children, teenage girls, premenopausal women, and the elderly (Hegarty 1995; Sanni et al. 2010). Deficiencies of some minerals may have serious implications on physical, psychological, and/or economic well‐being of humans (Hegarty 1995; Sanni et al. 2010). Soups selected had appreciable levels of iron content. The adult RDA for iron is 10 mg/day for men and 15 mg/day for women indicating that the selected soups will be able to meet the daily dietary iron requirements (Wardlaw 1999; Kayode et al. 2010). This higher amount of iron in these soup samples might be due to combinations of meats, fish, and other ingredients added to the soups. This corresponds to earlier reports that most Nigerian natural foods are rich in iron (Latunde‐Dada 1997). Openheimer (2000) reported that iron deficiency was extremely common in the developing world, with <50% of the world's population having some degree of deficient iron status based on a wide variety of tests. This corresponds to studies by Elemo et al. (2010b) on the iron status of premenopausal women in a Nigerian university. They reported that these women were at a very high risk of nutritional anemia. This could be attributed to their irregular diet, socioeconomic status, and consumption pattern. However, the presence of antinutrients such as phytatein food could reduce iron absorption and utilization in humans (Kayode et al. 2010).

#### Calcium

The results of calcium contents obtained for both freshly prepared and selected soup samples subjected to freeze–thaw cycles under different freezing and thawing conditions are revealed in Tables 6, 7, 8, 9. The freshly prepared soups had calcium values of 73.71 ± 0.04 mg/100 g (*Ogbono*), 147.53 ± 0.25 mg/100 g (*Ewedu*), 73.57 ± 0.18 mg/100 g (*Ila*), and 190.83 ± 0.08 mg/100 g (*Kuka*). The range of iron contents of the selected soup samples subjected to different frozen and thawing conditions were as follows: 73.28 ± 0.02–73.62 ± 0.05 mg/100 g (*Ogbono*), 141.51 ± 0.24–147.12 ± 0.24 mg/100 g (*Ewedu*), 68.11 ± 0.32–72.60 ± 0.37 mg/100 g (*Ila*), and 189.60 ± 0.39–190.79 ± 0.10 mg/100 g (*Kuka*). Freshly prepared *Kuka* soup was observed to have the highest concentration of calcium with freshly prepared *Ogbono* having the lowest concentration. Calcium is one of the macro minerals needed in highest amounts for proper body functions (Sanni et al. 2010). Calcium helps in regulating muscle contraction. It is also required by children and pregnant and lactating women for bones and teeth development (Olusanya 2008). The selected soups had relatively high levels but not sufficient to meet the adequate intake (AI) of calcium for adults (1000–1200 mg/day) and adolescence (1300 mg/day). Calcium deficiency is certainly a risk factor for osteoporosis in later life (Allen 2001). This makes supplementation very important. Flesh and sea foods are often included in these soups and also consumed with tuber or cereal‐based dishes such as cooked cassava, yam, plantain, rice, or maize‐based dishes thus improving the calcium level.

#### Sodium

Tables 6, 7, 8, 9 present the sodium contents obtained for both freshly prepared and selected soup samples subjected to freeze–thaw cycles under different freezing and thawing conditions. The freshly prepared soups had the sodium values of 160.21 ± 0.26 mg/100 g (*Ogbono*), 150.29 ± 0.05 mg/100 g (*Ewedu*), 152.48 ± 1.24 mg/100 g (*Ila*), and 231.72 ± 0.03 mg/100 g (*Kuka*). The range sodium contents of the selected soup samples subjected to different frozen and thawing conditions were as follows: 159.18 ± 0.03–159.93 ± 0.16 mg/100 g (*Ogbono*), 148.12 ± 0.08–149.90 ± 0.43 mg/100 g (*Ewedu*), 144.39 ± 0.14–152.24 ± 1.26 mg/100 g (*Ila*), and 230.83 ± 0.06–231.64 ± 0.07 mg/100 g (*Kuka*). Freshly prepared *Kuka* was observed to have a very high concentration of sodium while freshly prepared *Ewedu* had the lowest. It had been established that sodium is needed in highest amount for proper functioning of the body system (Sanni et al. 2010). Sodium is the major positive ion in the extracellular fluid and a key factor in retaining body water. All the soups analyzed had values within the RDA. Under the FDA food‐label ingredients, the daily value for sodium is 2400 mg (Greely 1997). High sodium content has been shown to contribute to hypertension in susceptible individuals, leading to increased calcium loss in urine (Wardlaw 1999). The ratio of sodium to potassium (Na/K) in the body is of great concern for prevention of high blood pressure. Na/K ratio <1 is recommended (FND, 2002). Hence, consumption of *Ogbono*,* Ewedu*,* Ila*, and *Kuka* soups may not be connected with high blood pressure disease since their Na/K ratio is <1.

#### Potassium

Tables 6, 7, 8, 9 show the potassium contents obtained for both freshly prepared and selected soup samples subjected to freeze–thaw cycles under different freezing and thawing conditions. The freshly prepared soups had the potassium value of 260.50 ± 0.50 mg/100 g (*Ogbono*), 206.30 ± 0.05 mg/100 g (*Ewedu*), 210.24 ± 0.27 mg/100 g (*Ila*), and 302.07 ± 0.06 mg/100 g (*Kuka*). The range of potassium contents of the selected soup samples subjected to different frozen and thawing conditions were as follows: 259.21 ± 0.04–260.03 ± 0.05 mg/100 g (*Ogbono*), 204.74 ± 0.28–206.16 ± 0.15 mg/100 g (*Ewedu*), 200.38 ± 0.97–210.04 ± 0.36 mg/100 g (*Ila*), and 301.16 ± 0.13–301.97 ± 0.07 mg/100 g (*Kuka*). A high concentration of potassium was observed in freshly prepared *Kuka* soups, with freshly prepared *Ewedu* having the lowest concentration.

Potassium is also among the macrominerals needed in highest amounts for proper body functions (Sanni et al. 2010). High amounts of potassium were observed in this study and the soups are expected to contribute to proper functioning of the body systems. High amount of potassium in the body was reported to increase iron utilization and it is beneficial to people taking diuretic to control hypertension and excessive excretion of potassium through the body fluid (HMSO, 1994, DuruMajesty et al. 2012). Deficiency in potassium leads to an irregular heartbeat, loss of appetite, and muscle cramps, but as stated earlier, these soups are often not consumed alone but with other food types which could improve the potassium level (Kayode et al. 2010).

#### Phosphorus

The phosphorus contents obtained for both freshly prepared and selected soup samples subjected to freeze–thaw cycles under different freezing and thawing conditions are presented in Tables 6, 7, 8, 9. The freshly prepared soups had the phosphorus values of 337.19 ± 0.07 mg/100 g (*Ogbono*), 312.43 ± 0.08 mg/100 g (*Ewedu*), 238.84 ± 1.15 mg/100 g (*Ila*), and 323.52 ± 0.50 mg/100 g (*Kuka*). The range of phosphorus contents of the selected soup samples subjected to different frozen and thawing conditions were as follows: 336.49 ± 0.07–337.13 ± 0.12 mg/100 g (*Ogbono*), 310.87 ± 0.27–312.29 ± 0.05 mg/100 g (*Ewedu*), 227.99 ± 0.30–237.37 ± 0.88 mg/100 g (*Ila*), and 321.76 ± 0.13–323.37 ± 0.07 mg/100 g (*Kuka*). Phosphorus was observed to be low in freshly prepared *Ila* compared to freshly prepared *Ogbono* which had a rather very high concentration.

Phosphorus works in conjunction with calcium to facilitate some roles of calcium in the body system (Dosunmu 1997; Turan et al. 2003). Good calcium and phosphorus ratio should be close to unity (Kayode et al. 2010). Although no disease is currently associated with an inadequate phosphorus intake, its deficiency may contribute to bone loss in elderly women (Wardlaw 1999). The selected soups had a very high level of phosphorus. This indicates that the soups can meet the daily requirements of phosphorus (RDA for adults is >700 mg/day) (Kayode et al. 2010). There were slight changes in all the minerals evaluated with respect to freeze–thaw cycles, packaging materials, and thawing conditions. This could be attributed to drip loss and dehydration that are associated with frozen storage (Sikorski and Kolakowski 2000). Similar result was reported by Arannilewa et al. (2005) for the effect of freezing periods on the mineral composition of tilapia fish. However, soups packaged in plastic containers and thawed under microwave‐thawing condition had the least mineral losses at every freeze–thaw cycle, when compared with others. This could be attributed to the nature of the packaging material and the rapid thawing effect of microwave as mentioned earlier.

### Effect of freezing and thawing conditions on the vitamin contents of some selected Nigerian soups

Tables 10, 11, 12, 13 present the effect of freezing and thawing conditions on the vitamins A, B, and E contents of *Ogbono*,* Ewedu*,* Ila*, and *Kuka* soups subjected to frozen storage. The freezing and thawing conditions significantly influenced all the determined properties at 95% confidence level when compared with freshly prepared soups. Vitamins are potent organic compounds found in certain foods and perform specific and vital functions in body chemistry (Paul and Pearson 2005). These vitamins differ from each other in physiological function, chemical structure, and their distribution in food. They are broadly divided into two categories: water‐soluble and fat‐soluble vitamins (Julie, 2003).

**Table 10:** Effect of freezing and thawing conditions on the vitamin contents of *Ogbono* soup

Sample	Vitamin A (mg/100 g)	Vitamin B (mg/100 g)	Vitamin E (mg/100 g)
Cycle 0			
O	39.92 ± 0.08^a^	36.42 ± 0.07^a^	74.00 ± 0.15^a^
Cycle 1			
AH	39.74 ± 0.01^cd^	36.25 ± 0.01^cd^	73.67 ± 0.03^cd^
AR	39.16 ± 0.01^k^	35.73 ± 0.01^k^	72.60 ± 0.02^k^
AM	39.58 ± 0.02^g^	36.11 ± 0.02^g^	73.37 ± 0.03^g^
PH	39.80 ± 0.01^b^	36.31 ± 0.01^b^	73.78 ± 0.02^b^
PR	39.58 ± 0.01^g^	36.11 ± 0.01^g^	73.37 ± 0.02^g^
PM	39.83 ± 0.03^b^	36.33 ± 0.02^b^	73.83 ± 0.05^b^
Cycle 2			
AH	39.56 ± 0.03^g^	36.09 ± 0.03^g^	73.33 ± 0.06^g^
AR	38.41 ± 0.02^n^	35.04 ± 0.02^n^	71.20 ± 0.04^n^
AM	39.24 ± 0.04^j^	35.80 ± 0.04^j^	72.74 ± 0.08^j^
PH	39.71 ± 0.02^de^	36.22 ± 0.02^de^	73.61 ± 0.03^de^
PR	39.24 ± 0.02^j^	35.80 ± 0.02^j^	72.74 ± 0.03^j^
PM	39.75 ± 0.02^c^	36.27 ± 0.02^c^	73.69 ± 0.04^c^
Cycle 3			
AH	39.41 ± 0.02^i^	35.95 ± 0.02^i^	73.05 ± 0.04^i^
AR	37.65 ± 0.02^o^	34.35 ± 0.02^o^	69.80 ± 0.04^o^
AM	38.93 ± 0.03^l^	35.52 ± 0.03^l^	72.17 ± 0.05^l^
PH	39.60 ± 0.02^fg^	36.13 ± 0.02^fg^	73.42 ± 0.04^fg^
PR	38.90 ± 0.03^l^	35.49 ± 0.02^l^	72.11 ± 0.05^l^
PM	39.69 ± 0.02^e^	36.21 ± 0.02^e^	73.58 ± 0.03^e^
Cycle 4			
AH	39.24 ± 0.02^j^	35.80 ± 0.02^j^	72.75 ± 0.04^j^
AR	36.93 ± 0.02^p^	33.69 ± 0.02^p^	68.45 ± 0.03^p^
AM	38.61 ± 0.03^m^	35.22 ± 0.03^m^	71.58 ± 0.05^m^
PH	39.49 ± 0.03^h^	36.33 ± 0.02^b^	73.20 ± 0.06^h^
PR	38.58 ± 0.02^m^	35.19 ± 0.02^m^	71.51 ± 0.04^m^
PM	39.63 ± 0.01^f^	36.15 ± 0.01^f^	73.46 ± 0.02^f^

Values are means of three replicates. Mean values having different superscripts within a column are significantly different (*P* < 0.05). PH, packaged in plastics but thawed with hot water; AH, packaged in aluminum but thawed with hot water; PM, packaged in plastics but thawed with microwave; AM, packaged in aluminum but thawed with microwave; AR, packaged in aluminum but thawed at room temperature; PR, packaged in plastics but thawed at room temperature.

**Table 11:** Effect of freezing and thawing conditions on the vitamin contents of *Ewedu* soup

Sample	Vitamin A (mg/100 g)	Vitamin B (mg/100 g)	Vitamin E (mg/100 g)
Cycle 0			
E	31.54 ± 0.05^a^	10.75 ± 0.02^a^	30.36 ± 0.05^a^
Cycle 1			
AH	31.35 ± 0.01^c^	10.69 ± 0.00^c^	30.18 ± 0.01^c^
AR	31.13 ± 0.02^ef^	10.61 ± 0.01^ef^	29.97 ± 0.02^ef^
AM	31.09 ± 0.05^f^	10.60 ± 0.02^f^	29.93 ± 0.05^f^
PH	31.20 ± 0.02^de^	10.64 ± 0.01^de^	30.04 ± 0.02^de^
PR	31.24 ± 0.03^d^	10.65 ± 0.01^a^	30.08 ± 0.03^a^
PM	31.49 ± 0.01^a^	10.74 ± 0.00^a^	30.32 ± 0.01^a^
Cycle 2			
AH	31.13 ± 0.02^ef^	10.61 ± 0.01^ef^	29.97 ± 0.02^ef^
AR	30.89 ± 0.25^gh^	10.53 ± 0.09^gh^	29.74 ± 0.24^gh^
AM	30.54 ± 0.03^j^	10.41 ± 0.01^j^	29.40 ± 0.03^j^
PH	30.87 ± 0.01^h^	10.52 ± 0.00^h^	29.72 ± 0.01^h^
PR	30.99 ± 0.03^g^	10.56 ± 0.01^g^	29.83 ± 0.03^g^
PM	31.47 ± 0.02^ab^	10.73 ± 0.01^ab^	30.29 ± 0.02^ab^
Cycle 3			
AH	30.93 ± 0.04^gh^	10.54 ± 0.01^gh^	29.78 ± 0.04^gh^
AR	30.37 ± 0.03^k^	10.35 ± 0.01^k^	29.23 ± 0.03^k^
AM	30.09 ± 0.02^m^	10.26 ± 0.01^m^	28.97 ± 0.02^m^
PH	30.55 ± 0.04^j^	10.41 ± 0.01^j^	29.41 ± 0.04^j^
PR	30.75 ± 0.02^i^	10.48 ± 0.01^i^	29.60 ± 0.02^i^
PM	31.44 ± 0.03^a–c^	10.72 ± 0.01^a–c^	30.26 ± 0.03^a–c^
Cycle 4			
AH	30.74 ± 0.01^i^	10.48 ± 0.00^i^	29.60 ± 0.01^i^
AR	29.95 ± 0.05^n^	10.21 ± 0.02^n^	28.83 ± 0.05^n^
AM	29.54 ± 0.06^o^	10.07 ± 0.02^o^	28.44 ± 0.05^o^
PH	30.22 ± 0.03^l^	10.30 ± 0.01^l^	29.09 ± 0.03^l^
PR	30.48 ± 0.02^j^	10.39 ± 0.01^j^	29.35 ± 0.02^j^
PM	31.39 ± 0.02^bc^	10.70 ± 0.01^bc^	30.22 ± 0.02^bc^

Values are means of three replicates. Mean values having different superscripts within a column are significantly different (*P* < 0.05). PH, packaged in plastics but thawed with hot water; AH, packaged in aluminum but thawed with hot water; PM, packaged in plastics but thawed with microwave; AM, packaged in aluminum but thawed with microwave; AR, packaged in aluminum but thawed at room temperature; PR, packaged in plastics but thawed at room temperature.

**Table 12:** Effect of freezing and thawing conditions on the vitamin contents of *Ila* soup

Sample	Vitamin A (mg/100 g)	Vitamin B (mg/100 g)	Vitamin E (mg/100 g)
Cycle 0			
I	59.88 ± 0.27^a^	14.08 ± 0.06^a^	90.24 ± 0.40^a^
Cycle 1			
AH	59.93 ± 0.08^a^	14.10 ± 0.02^a\^	90.32 ± 0.12^a^
AR	58.51 ± 0.16^fg^	13.76 ± 0.04^fg^	88.17 ± 0.24^fg^
AM	59.11 ± 0.19^cd^	13.90 ± 0.04^cd^	89.08 ± 0.29^cd^
PH	59.34 ± 0.17^bc^	13.96 ± 0.04^bc^	89.42 ± 0.25^bc^
PR	59.38 ± 0.31^bc^	13.96 ± 0.07^bc^	89.48 ± 0.47^bc^
PM	59.80 ± 0.10^a^	14.07 ± 0.02^a^	90.12 ± 0.15^a^
Cycle 2			
AH	59.49 ± 0.16^b^	13.99 ± 0.04^b^	89.65 ± 0.25^b^
AR	57.55 ± 0.07^i^	13.53 ± 0.01^i^	86.72 ± 0.10^i^
AM	58.58 ± 0.06^fg^	13.78 ± 0.01^fg^	88.29 ± 0.09^fg^
PH	58.88 ± 0.11^de^	13.85 ± 0.03^de^	88.73 ± 0.16^de^
PR	58.74 ± 0.22^ef^	13.81 ± 0.05^ef^	88.52 ± 0.33^ef^
PM	59.49 ± 0.14^b^	13.99 ± 0.03^b^	89.66 ± 0.21^b^
Cycle 3			
AH	59.39 ± 0.08^bc^	13.97 ± 0.02^bc^	89.50 ± 0.12^bc^
AR	56.22 ± 0.07^j^	13.22 ± 0.02^j^	84.73 ± 0.11^j^
AM	58.06 ± 0.08^h^	13.66 ± 0.02^h^	87.50 ± 0.12^h^
PH	58.57 ± 0.10^fg^	13.78 ± 0.02^fg^	88.27 ± 0.15^fg^
PR	58.45 ± 0.18^g^	13.75 ± 0.04^g^	88.09 ± 0.27^g^
PM	59.41 ± 0.09^b^	13.97 ± 0.02^b^	89.53 ± 0.13^b^
Cycle 4			
AH	59.27 ± 0.09^bc^	13.94 ± 0.02^bc^	89.31 ± 0.14^bc^
AR	54.93 ± 0.23^k^	12.92 ± 0.05^k^	82.79 ± 0.34^k^
AM	57.45 ± 0.08^i^	13.51 ± 0.02^i^	86.57 ± 0.12^i^
PH	58.39 ± 0.06^g^	13.73 ± 0.01^g^	88.00 ± 0.10^g^
PR	57.89 ± 0.05^h^	13.61 ± 0.01^h^	87.23 ± 0.08^h^
PM	59.30 ± 0.16^bc^	13.95 ± 0.04^bc^	89.37 ± 0.24^bc^

Values are means of three replicates. Mean values having different superscripts within a column are significantly different (*P* < 0.05). PH, packaged in plastics but thawed with hot water; AH, packaged in aluminum but thawed with hot water; PM, packaged in plastics but thawed with microwave; AM, packaged in aluminum but thawed with microwave; AR, packaged in aluminum but thawed at room temperature; PR, packaged in plastics but thawed at room temperature.

**Table 13:** Effect of freezing and thawing conditions on the vitamin contents of *Kuka* soup

Sample	Vitamin A (mg/100 g)	Vitamin B (mg/100 g)	Vitamin E (mg/100 g)
Cycle 0			
K	43.11 ± 0.03^a^	24.08 ± 0.02^a^	40.12 ± 0.03^a^
Cycle 1			
AH	42.71 ± 0.01^c^	23.86 ± 0.01^c^	39.74 ± 0.01^c^
AR	41.91 ± 0.26^g^	23.41 ± 0.15^g^	39.00 ± 0.24^g^
AM	42.54 ± 0.03^d^	23.76 ± 0.01^g^	39.59 ± 0.02^g^
PH	42.91 ± 0.02^b^	23.97 ± 0.01^g^	39.93 ± 0.02^g^
PR	42.33 ± 0.01^e^	23.65 ± 0.01^g^	39.39 ± 0.01^g^
PM	42.76 ± 0.02^c^	23.89 ± 0.01^c^	39.79 ± 0.02^c^
Cycle 2			
AH	42.31 ± 0.02^e^	23.64 ± 0.01^e^	39.38 ± 0.02^e^
AR	41.02 ± 0.02^k^	22.91 ± 0.01^k^	38.17 ± 0.02^k^
AM	41.99 ± 0.03^g^	23.46 ± 0.01^g^	39.08 ± 0.02^g^
PH	42.70 ± 0.02^c^	23.85 ± 0.01^c^	39.73 ± 0.02^c^
PR	41.56 ± 0.02^i^	23.22 ± 0.01^i^	38.67 ± 0.02^i^
PM	42.44 ± 0.02^d^	23.71 ± 0.01^d^	39.49 ± 0.02^d^
Cycle 3			
AH	41.93 ± 0.02^g^	23.42 ± 0.01^g^	39.02 ± 0.02^g^
AR	39.94 ± 0.08^m^	22.31 ± 0.04^m^	37.16 ± 0.07^m^
AM	41.45 ± 0.02^j^	23.15 ± 0.01^j^	38.57 ± 0.02^j^
PH	42.48 ± 0.05^d^	23.73 ± 0.03^d^	39.53 ± 0.05^d^
PR	40.83 ± 0.02^l^	22.81 ± 0.01^l^	37.99 ± 0.02^l^
PM	42.10 ± 0.03^f^	23.52 ± 0.01^f^	39.18 ± 0.02^f^
Cycle 4			
AH	41.52 ± 0.04^ij^	23.19 ± 0.02^ij^	38.64 ± 0.03^ij^
AR	38.87 ± 0.08^n^	21.72 ± 0.05^n^	36.17 ± 0.08^n^
AM	40.93 ± 0.01^k^	22.86 ± 0.01^n^	38.09 ± 0.01^n^
PH	42.31 ± 0.02^e^	23.63 ± 0.01^e^	39.37 ± 0.02^e^
PR	40.02 ± 0.02^m^	22.36 ± 0.01^m^	37.24 ± 0.02^m^
PM	41.81 ± 0.02^h^	23.36 ± 0.01^h^	38.91 ± 0.02^h^

Values are means of three replicates. Mean values having different superscripts within a column are significantly different (*P* < 0.05). PH, packaged in plastics but thawed with hot water; AH, packaged in aluminum but thawed with hot water; PM, packaged in plastics but thawed with microwave; AM, packaged in aluminum but thawed with microwave; AR, packaged in aluminum but thawed at room temperature; PR, packaged in plastics but thawed at room temperature.

#### Vitamin A

Tables 10, 11, 12, 13 present the vitamin A values obtained for both freshly prepared soups and soups samples subjected to freeze–thaw cycles under different freezing and thawing conditions. The freshly prepared soups had vitamin A values of 39.92 ± 0.08 mg/100 g (*Ogbono*), 31.54 ± 0.05 mg/100 g (*Ewedu*), 59.88 ± 0.27 mg/100 g (*Ila*), and 43.11 ± 0.03 mg/100 g (*Kuka*). While the vitamin A values of the selected soup samples subjected to different frozen and thawing conditions ranged from 36.93 ± 0.02 to 39.83 ± 0.03 mg/100 g (*Ogbono*), 29.54 ± 0.06 to 31.49 ± 0.01 mg/100 g (*Ewedu*), 54.93 ± 0.23 to 59.30 ± 0.16 mg/100 g (*Ila*), and 38.87 ± 0.08 to 42.91 ± 0.02 mg/100 g (*Kuka*). It was generally observed that the vitamin A (retinol) content of the soups slightly decreased during freeze storage as they were significantly influenced by the nature of the packaging materials and thawing conditions. Retention of the vitamin A contents under frozen storage might be due to absence of light and air that could cause oxidation which could lead to a considerable depletion of the vitamin A (Eze and Akubor 2012). Slight decrease in the vitamin A contents might be as a result of heat application and thawing duration. Vitamin A (retinol) are known as endogenous antioxidants that can act as scavengers of free radicals, so that protection against the very early stages of lipid oxidation would be favored (Jensen et al. 1998). It is needed for maintenance of skin, mucus membrane, bones, teeth, hair, vision, and reproduction (DuruMajesty et al. 2012). The protective effects of carotenoids (vitamin A precursors) against serious disorders such as heart disease, cancer, and degenerative eye disease had been recognized (Ejoh et al. 2007). The consequences of vitamin A deficiency (VAD) include night blindness, Bitot's spot, corneal xerosis, and corneal scars or ulcers. Other serious consequences have been shown to include increased morbidity and mortality of infants, children, and pregnant women, poor growth of children, and susceptibility to anemia through interface with iron transport and utilization for hemoglobin synthesis (Hart et al. 2005). High amounts of vitamin A were obtained in this study. The observed *β*‐carotene content of the soups was able to meet the RDA for *β*‐carotene estimated at 6 mg/day (10,000 IU/day) (Wardlaw 1999; Okeke and Eze 2006) and they are expected to contribute to proper functioning of the body systems. Carotenoids are susceptible to oxidation when they are exposed to light, oxygen, warm temperature, enzyme, and moisture. The conditions encountered with thawing such as exposure to light, temperature, air, and loss of moisture might have been involved in the destruction of carotenoid. During thawing process, catalytic enzyme such as lipoxygenase might be activated in the soups (Park 1987; Paul and Pearson 2005). Lipoxygenase is relatively thermostable with pH optimum of 6.5. It is capable of forming reactive radicals which can oxidize carotenoid, chlorophyll, and other substances (Park 1987; Paul and Pearson 2005). Microwave thawing of soups in plastic containers retained more vitamin A than other thawing methods. This might be due to shorter thawing time and other conditions mentioned above. The reduction in *β*‐carotene content of soups after thawing corresponds to various reports. Anjum et al. (2008) reported a pronounced reduction in *β*‐carotene content of selected Indian vegetables during processing. Elemo et al. (2011) established that processing methods caused significant decrease (*P* < 0.05) in the *β*‐carotene of the green leafy vegetables and combination of the leafy vegetables with other foodstuffs was recommended to satisfactorily meet the RDA.

#### Vitamin B

Tables 10, 11, 12, 13 present the vitamin B values obtained for both freshly prepared soups and soups samples subjected to freeze–thaw cycles under different freezing and thawing conditions. The freshly prepared soups had the vitamin B values of 36.42 ± 0.07 mg/100 g (*Ogbono*), 10.75 ± 0.02 mg/100 g (*Ewedu*), 14.08 ± 0.06 mg/100 g (*Ila*), and 24.08 ± 0.02 mg/100 g (*Kuka*). While the vitamin B values of the selected soup samples subjected to different frozen and thawing conditions ranged from 36.33 ± 0.02 to 36.33 ± 0.02 mg/100 g (*Ogbono*), 10.07 ± 0.02 to 10.74 ± 0.00 mg/100 g (*Ewedu*), 12.92 ± 0.05 to 14.10 ± 0.02 mg/100 g (*Ila*), and 21.72 ± 0.05 to 23.97 ± 0.01 mg/100 g (*Kuka*). Freshly prepared *Ogbono* soup was observed to have the highest concentration of vitamin B with freshly prepared *Ewedu* having the lowest concentration. Vitamin B1 (thiamine) is needed for nervous system and helps in releasing energy from carbohydrate (Paul and Pearson 2005). There was slight reduction in the vitamin B evaluated with respect to freeze–thaw cycles, packaging materials, and thawing conditions. Factors influencing the vitamin B stability of frozen foods include the temperature of the freezing unit and its range of fluctuation, the length of storage, the size of the cut, the thawing method, and the packaging method. Ideally, a temperature of at least −18°C should be used to store both animal and vegetable foods (Severi et al. 1997). Fluctuations in the freezing temperature might be responsible for significant losses of vitamins in soups (Severi et al. 1997). The length of storage affects significantly the retention of vitamins and losses of thiamine tends to increase as the freeze–thaw cycle increased. The thawing duration significantly influence the vitamin B content of the soups, soups with shorter thawing time had high vitamin B retention ability. This might be due to vitamin B susceptibility to heat damage (Aubourg 2001). The selected soups had a high level of vitamin B. This indicates that the soups can meet the daily requirements of vitamin B (RDA for adults is >63 *μ*g/day) (Okeke and Eze 2006).

#### Vitamin E

The results of vitamin E content obtained for both freshly prepared and selected soup samples subjected to freeze–thaw cycles under different freezing and thawing conditions are revealed in Tables 10, 11, 12, 13. The freshly prepared soups had the vitamin E values of 74.00 ± 0.15 mg/100 g (*Ogbono*), 30.36 ± 0.05 mg/100 g (*Ewedu*), 90.24 ± 0.40 mg/100 g (*Ila*), and 40.12 ± 0.03 mg/100 g (*Kuka*). While the vitamin B values of the selected soup samples subjected to different frozen and thawing conditions ranged from 68.45 ± 0.03 to 73.83 ± 0.05 mg/100 g (*Ogbono*), 28.44 ± 0.05 to 30.32 ± 0.01 mg/100 g (*Ewedu*), 82.79 ± 0.34 to 90.32 ± 0.12 mg/100 g (*Ila*), and 36.17 ± 0.08 to 39.79 ± 0.02 mg/100 g (*Kuka*). It was observed that the soups contained vitamin E in moderate concentrations and the variation in the loss of vitamin E of the soups under investigation demonstrated the differences in vulnerabilities of the selected soup to spoilage at every freeze–thaw cycle. Vitamins become important when their functions are considered in the body. Vitamin E (Tocopherol) acts as antioxidants that protects cell wall and aids in reproduction (Julie, 2003; Wardlaw and Kessel 2002). The observed tocopherol content of the soups would be able to meet the RDA for tocopherol estimated at 15 mg/day (Okeke and Eze 2006). The vitamin E can act as an anticoagulant and may increase the risk of bleeding problems and many agencies have set an upper tolerable intake level (UL) for vitamin E at 1000 mg/day (DuruMajesty et al. 2012). Also, the European Food Safety Authority by its Scientific Committee on Food (SCF) has set a tolerable upper intake level (UL) of vitamin E for adult at 300 mg/day (Okeke and Eze 2006). However, the vitamin E reported in this study was within the safe limit and will not constitute any health hazard.

## Conclusions

The result of the investigation shows that *Ila*,* Ewedu*,* Ogbono*, and *Kuka* soups are good sources of vitamins and minerals. Deterioration increased as freeze–thaw cycle increased and that the nutritional quality of the soups was best before subjection to freeze–thaw cycles. The nutritional quality of *Ila*,* Ewedu*,* Ogbono*, and *Kuka* soups depends on container type, freeze–thaw cycles, and thawing methods. Microwave‐thawed plastic soups had limited nutritional losses when compared with other thawing methods, and should not be extended beyond the third cycle because of increasing reduction in fat and protein, indicating deterioration.

## Conflict of Interest

None declared.
